# High-quality draft genome sequences of *Pseudomonas guariconensis* ABJ_B2_1 and *Pseudomonas shirazica* ABJ_A22_2_1

**DOI:** 10.1128/mra.00194-25

**Published:** 2025-06-12

**Authors:** Ahmed Olowo-okere, E. Skiebe, G. Wilharm

**Affiliations:** 1 Department of Pharmaceutical Microbiology and Biotechnology, Faculty of Pharmaceutical Sciences, University of Abuja 99399, Abuja, Federal Capital Territory, Nigeria; 2 Project Group P2, Robert Koch-Institute 235859 , Wernigerode, Saxony-Anhalt, Germany; Loyola University Chicago, Chicago, Illinois, USA

**Keywords:** *Pseudomonas guariconensis*, *Pseudomonas shirazica*, draft genome, soil, Nigeria

## Abstract

We report the draft genome sequences of *Pseudomonas guariconensis* ABJ_B2_1 and *Pseudomonas shirazica* ABJ_A22_2_1, isolated from soil in Gwagwalada, Nigeria. The genomes were 4.98 Mb (40 contigs) and 5.71 Mb (112 contigs) in size, with GC contents of 62.68% and 62.63%, respectively.

## ANNOUNCEMENT

The genus *Pseudomonas* is a large and diverse family of non-fermentative, gram-negative bacilli. *Pseudomonas* species possess some of the largest genomes among gram-negative bacteria, harboring a diverse repertoire of genes that support their metabolic versatility and ecological adaptation (1). Members of this genus have been isolated from a wide range of environments, including clinical and environmental sources (1, 2).

Despite their ecological and clinical significance, there are limited genomic data on circulating *Pseudomonas* strains in Nigeria. In this study, we report the high-quality draft genome sequences of *Pseudomonas guariconensis* ABJ_B2_1 and *Pseudomonas shirazica* ABJ_A22_2_1, two soil-derived isolates from Nigeria.

Approximately 30 g of soil was collected at a depth of 20 cm using a sterile spatula. Soil type and coordinates are detailed in Table 1. One gram of each sample was pre-enriched in 10 mL mineral salt medium supplemented with 0.2% sodium acetate for 5 h, then plated on CHROMagar Acinetobacter (without MDR supplement) and incubated at 37°C overnight as previously described (3). Colonies displaying distinct pigmentation and morphology atypical of *Acinetobacter* species were purified on Columbia agar supplemented with 5% sheep blood and selected for further characterization.

**TABLE 1:** Genomic features, virulence, and antibiotic-resistant determinants identified in *P. guariconensis* ABJ_B2_1 and *P. shirazica* ABJ_A22_2_1

Feature	*P. guariconensis* ABJ_B2_1	*P. shirazica* ABJ_A22_2_1
Accession number	JBJDIX000000000	JBJFLQ000000000
Reads accession	SRR32940689	SRR32929196
Read counts	5,536,216	6,639,242
Average read length	277	265
Sampling points description	Exposed soil in a rock crevice	Cattle and goats’ sheds
Geographical coordinate	8°58′45″N 7°12′34″E	9°06′19″N 7°13′08″E
Average nucleotide identity (%)	97.42	97.05
Genome coverage (×)	308.0	307.9
Completeness (%)	100	100
Contamination (%)	0.37	0.32
Genome size (bp)	4,983,367	5,712,735
GC content (%)	62.68	62.63
Contig *N*_50_ (bp)	262,383	159,215
Total contigs	40	112
Max contig length (bp)	554,137	445,486
Total coding sequences	4,549	5,160
Total genes	4,652	5,284
RNA genes	74	91
Pseudogenes	71	72
Predicted virulence genes	*pvdM, pvdS, mbtH-like, algU, algW, alg8, algI, algA, waaF, waaG, motA, fleQ, flhA, fleN, flgB, flgC, flgG, flgH, flgI, fliG, fliI, fliM, fliN, fliP, pilH*	*pvdH, pvdS, mbtH-like, algA, algI, alg8, algD, algU, algW, algB, waaF, waaG, motC, fleQ, flhA, fleN, flgH, flgI, flgG, fliG, fliI, fliM, fliN, fliP, fliQ, pilH*
Predicted resistance genes	*MexF, MexK, MexB, MexW*	*MexF, MexK, MexB, MexW, CpxR*

Genomic DNA was extracted using the MasterPure DNA Purification Kit (Epicentre) and quantified using the Invitrogen Qubit 4 fluorometer according to the manufacturer’s instructions. Paired-end libraries were prepared using the Nextera XT DNA Library Prep Kit and sequenced on an Illumina NextSeq platform. *De novo* genome assembly was performed using Shovill version 1.1.0 (https://github.com/tseemann/shovill) with the default SPAdes version 4.0.0 genome assembler and --trim option (4). The completeness and contamination of the assemblies were assessed using CheckM2 version 1.0.2 and QUAST version 5.2.0 (5, 6). To confirm the taxonomic classification of the strains, average nucleotide identity (ANI) values were determined between the genomes of strains and the genomes of their respective type strains using fastANI version 1.33 (7). Genome annotation was performed using the NCBI Prokaryotic Genome Annotation Pipeline version 6.8 (8). Antibiotic resistance genes and virulence factors were identified using ABRicate version 1.0.1 against ResFinder and VFDB databases (14 April 2025) (9–11). All bioinformatics analyses were performed using default parameters for each software tool.

The genome of *P. guariconensis* ABJ_B2_1 (ASM4490304v1) comprises 40 contigs, with a total length of 4,983,367 bp, an *N*_50_ of 262,383 bp, and a GC content of 62.68%. On the other hand, the *P. shirazica* ABJ_A22_2_1 (ASM4503255v1) genome consists of 112 contigs, with a total length of 5,712,735 bp, an *N*_50_ of 159,215 bp, and a GC content of 62.63%. Genome annotation identified 4,652 and 5,284 protein-coding genes in *P. guariconensis* ABJ_B2_1 and *P. shirazica* ABJ_A22_2_1, respectively, along with 74 and 91 RNA genes (Table 1). ANI analysis revealed 97.05% nucleotide identity between *P. shirazica* ABJ_A22_2_1 and *P. shirazica* A28^T^ (ASM1516833v1) and 97.42% identity between *P. guariconensis* ABJ_B2_1 and *P. guariconensis* NY14324^T^ (ASM4082203v1). The predicted antibiotic resistance genes and virulence genes in the genomes are listed in Table 1.

This report provides valuable insights into the genomic features and potential ecological roles of these environmental *Pseudomonas* species.

## Data Availability

The draft genomes of *P. guariconensis* ABJ_B2_1 and *P. shirazica* ABJ_A22_2_11 have been deposited at NCBI/GenBank under the BioProject accessions PRJNA1183762 and PRJNA1183992. Genome accessions are available in Table 1.

## References

[1] Saati-Santamaría Z, Baroncelli R, Rivas R, García-Fraile P. 2022. Comparative genomics of the genus Pseudomonas reveals host- and environment-specific evolution. Microbiol Spectr 10:e0237022. doi:10.1128/spectrum.02370-22 36354324 PMC9769992

[2] Olowo-okere A, Ibrahim YKE, Ehinmidu JO, Mohammed Y, Nabti LZ, Olayinka BO. 2020. Emergence of VIM metallo-β-lactamase among carbapenem-resistant Pseudomonas species in Northwest Nigeria. Gene Rep 21:100877. doi:10.1016/j.genrep.2020.100877

[3] Yusuf I, Skiebe E, Wilharm G. 2023. Evaluation of CHROMagar Acinetobacter and MacConkey media for the recovery of Acinetobacter baumannii from soil samples. Lett Appl Microbiol 76:vac051. doi:10.1093/lambio/ovac051 36695425

[4] Bankevich A, Nurk S, Antipov D, Gurevich AA, Dvorkin M, Kulikov AS, Lesin VM, Nikolenko SI, Pham S, Prjibelski AD, Pyshkin AV, Sirotkin AV, Vyahhi N, Tesler G, Alekseyev MA, Pevzner PA. 2012. SPAdes: a new genome assembly algorithm and its applications to single-cell sequencing. J Comput Biol 19:455–477. doi:10.1089/cmb.2012.0021 22506599 PMC3342519

[5] Gurevich A, Saveliev V, Vyahhi N, Tesler G. 2025. QUAST: Quality Assessment Tool for Genome Assemblies | Bioinformatics | Oxford Academic. Available from: https://academic.oup.com/bioinformatics/article/29/8/1072/228832 10.1093/bioinformatics/btt086 PMC3624806 23422339

[6] Chklovski A, Parks DH, Woodcroft BJ, Tyson GW. 2023. CheckM2: a rapid, scalable and accurate tool for assessing microbial genome quality using machine learning. Nat Methods 20:1203–1212. doi:10.1038/s41592-023-01940-w 37500759

[7] Jain C, Rodriguez-R LM, Phillippy AM, Konstantinidis KT, Aluru S. 2018. High throughput ANI analysis of 90K prokaryotic genomes reveals clear species boundaries. Nat Commun 9:5114. doi:10.1038/s41467-018-07641-9 30504855 PMC6269478

[8] 2025. NCBI prokaryotic genome annotation pipeline. Nucleic Acids Research. Available from: https://academic.oup.com/nar/article/44/14/6614/2468204 10.1093/nar/gkw569 PMC5001611 27342282

[9] Seemann T. 2023. Abricate: Mass screening of contigs for antimicrobial and virulence genes. https://github.com/tseemann/abricate.

[10] Alcock BP, Huynh W, Chalil R, Smith KW, Raphenya AR, Wlodarski MA, Edalatmand A, Petkau A, Syed SA, Tsang KK, et al.. 2023. CARD 2023: expanded curation, support for machine learning, and resistome prediction at the comprehensive antibiotic resistance database. Nucleic Acids Res 51:D690–D699. doi:10.1093/nar/gkac920 36263822 PMC9825576

[11] Florensa AF, Kaas RS, Clausen P, Aytan-Aktug D, Aarestrup FM. 2025. ResFinder – an open online resource for identification of antimicrobial resistance genes in next-generation sequencing data and prediction of phenotypes from genotypes. Oxford academic. Available from: https://academic.oup.com/jac/article/75/12/3491/5890997 10.1099/mgen.0.000748 PMC8914360 35072601

